# Circulating tumor cell is a common property of brain glioma and promotes the monitoring system

**DOI:** 10.18632/oncotarget.11114

**Published:** 2016-08-08

**Authors:** Faliang Gao, Yong Cui, Haihui Jiang, Dali Sui, Yonggang Wang, Zhongli Jiang, Jizong Zhao, Song Lin

**Affiliations:** ^1^ Department of Neurosurgery, Beijing Tiantan Hospital, Capital Medical University, Beijing, China; ^2^ China National Clinical Research Center for Neurological Diseases, Beijing, China; ^3^ Institute for Brain Disorders and Beijing Key Laboratory of Brian Tumor, Beijing, China; ^4^ Department of Neurosurgery, First Hospital of Tsinghua University, Beijing, China

**Keywords:** glioma, circulating tumor cell (CTC), biomarker, monitor, radionecrosis

## Abstract

Brain glioma is the most common primary intracranial tumor characterized by dismal prognosis and frequent recurrence, yet a real-time and reliable biological approach to monitor tumor response and progression is still lacking. Recently, few studies have reported that circulating tumor cells (CTCs) could be detected in glioblastoma multiform (GBM), providing the possibility of its application in brain glioma monitoring system. But its application limits still exist, because the detection rate of CTCs is still low and was exclusively limited to high- grade gliomas. Here, we adopted an advanced integrated cellular and molecular approach of SE-iFISH to detect CTCs in the peripheral blood (PB) of patients with 7 different subtypes of brain glioma, uncovering the direct evidences of glioma migration. We identified CTCs in the PB from 24 of 31 (77%) patients with glioma in all 7 subtypes. No statistical difference of CTC incidence and count was observed in different pathological subtypes or WHO grades of glioma. Clinical data revealed that CTCs, to some extent, was superior to MRI in monitoring the treatment response and differentiating radionecrosis from recurrence of glioma. Conclusively, CTCs is a common property of brain gliomas of various pathological subtypes, which has provided an ultimate paradox for the hypothesis “soil and seed”. It can be used to monitor the microenvironment of gliomas dynamically, which will be a meaningful complement to radiographic imaging.

## INTRODUCTION

Gliomas are the most common primary intracranial tumor, representing 81% of malignant brain tumors [1]. In spite of the currently multimodal treatments, including surgery, chemotherapy and radiotherapy, the expectancy of survival time of glioma is still dismal [2]. The current standard for measuring the effects of treatment in gliomas is the application of the Response Assessment in Neuro-Oncology (RANO) criteria based on the radiological appearance of tumor on MRI [3]. But there is an unresolved problem-radionecrosis, a treatment-related response of brain tissue to radiation, in the radiological presentation of glioma, which could mimic true tumor progression [4]. Fortunately, liquid biopsy, a promising, noninvasive mean to evaluate the status of glioma, may potentially help in guiding patient treatment and management in the future [5].

Circulating tumor cells (CTCs), one of the most rewarding component in the collection of liquid biopsy, can potentially be used as significant biomarker, which have been validated in diverse types of solid tumors including lung, melanoma, osteosarcoma, pheochromocytoma, and parathyroid, etc. [5, 6]. In the past decades, the application of CTC in brain glioma has not been valued because although the high malignancy and invasiveness of glioma, very few case with extracranial metastases has been observed [7, 8]. Among all the proposed explanations for this phenomenon, the well-recognized viewpoint is that the special microenvironment of brain has limited the migration of glioma cells into circulations [9], hindering CTC application in monitoring brain glioma before. This misconception that brain glioma cells could never get into the blood, however, has been challenged in recent two years. In 2014, researchers have firstly found CTCs in the peripheral blood (PB) of patients with GBM, and declared that CTC is the “intrinsic property” of GBM biology [10]. Concerning the methodological deficiencies in previous studies, the incidence of CTCs, to some extent, is still very low, and the current results were exclusively limited to high-grade gliomas.

Recently we noticed that Ge et al. projected a novel integrated method to detect the CTCs based on the aneuploidy of chromosome 8 examination by CEP8-FISH, which has greatly improved the positive detection rate of CTCs in many types of tumors, especially in those patients with negative expression of tumor cell markers in their blood [6]. For example, it was reported that CK18, the dual epithelial marker and tumor biomarker, was positive in only 14% of lung and 24% of esophageal CTCs, respectively. It is well known that chromosome polyploidy is the common characteristics of tumor cells [11, 12] and it has been also proposed that aneuploidy could contribute to, or even drives, tumor development [12]. Furthermore, studies have demonstrated chromosome 8 aneuploidy in many solid tumors [13–18], providing the feasibility of CTCs detection based on aneuploidy chromosome. Although the expression and clinical significance of chromosome 8 in brain glioma have rarely discussed before, based on the advance in experiment technology, we speculate that CTCs could be found in all pathology subtypes of gliomas, not only the intrinsic property of GBM, and therefore is of great value in clinical application.

With this regard, in the present study, 31 patients with 7 different pathologic entities (grade II-IV) of primary gliomas were enrolled. CTC incidence and count in PB of patients with glioma was detected and comparison between different grades was launched. Next, we further investigated the effect of clinical intervention on CTC counts in gliomas. To better interpret the clinical application of CTCs, we explored its significance in distinguishing tumor recurrence from radiation necrosis. To our knowledge, this is the most systematic and comprehensive report on CTCs in different types of brain gliomas, and we believe that it could provide a new perspective towards research of CTCs.

## RESULTS

### Detection of polyploidy chromosome 8 in gliomas

**Figure 1:**
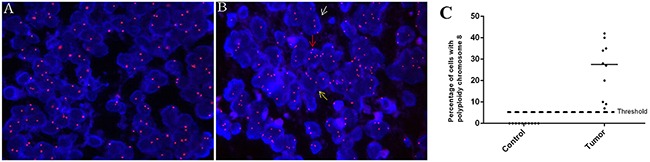
Detection of polyploidy chromosome 8 in glioma **A-B.** The status of chromosome 8 ploidy examined by FISH (orange, 10×100) in cells of a control and tumor specimen respectively. White, yellow and red arrow: a cell with triploid, tetraploid and more than 5 copies of chromosome 8 respectively. **C.** Comparison of cell enumeration with polyploidy chromosome 8 in control and tumor group.

To investigate the feasibility of detecting CTC in blood by CEP8-FISH, we firstly investigate the chromosome 8 polyploidy in brain glioma specimens. Figure 1A and 1B showed the status of cells with chromosome 8 ploidy in a control (Figure 1A) and glioma specimen (Figure 1B) examined by FISH. In a tumor specimen, cells were found with polyploidy chromosome 8, including triploid, tetraploid and more than 5 copies. The percentage of cells with polyploidy chromosome 8 in all 20 specimens was showed in Figure 1C. The results showed that chromosome 8 polyploidy cells generally existed in these 10 glioma specimens, compared to the results of control group. In control group, the mean percentage of cells with polyploidy chromosome 8 was 1.2%±1.31, contributing to the interpretation threshold 5.1% (Figure 1C).

### Characteristics of CTCs in PB of glioma patients

**Figure 2:**
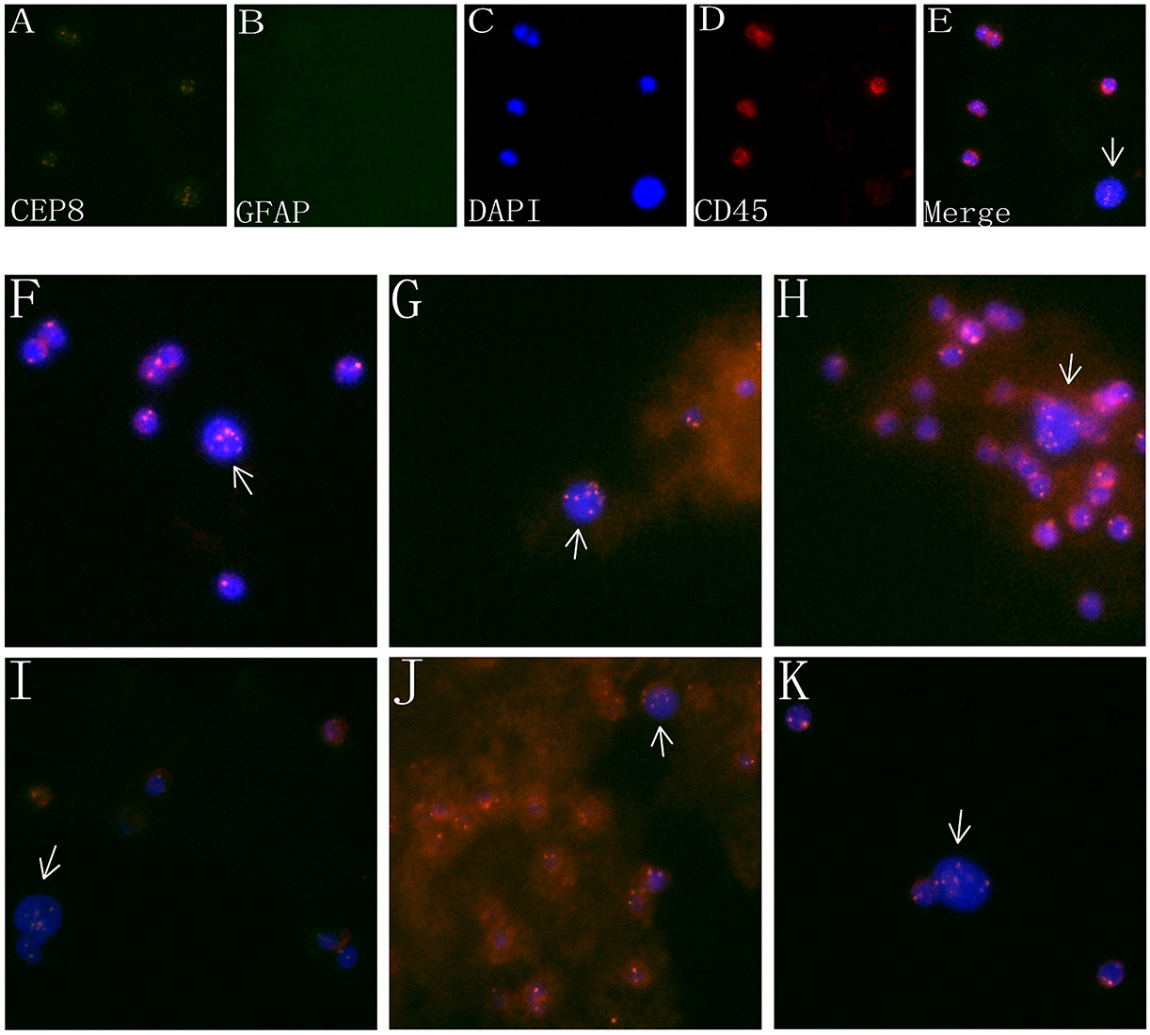
Characteristics of CTCs in the PB of glioma patients **A-D.** Images of a CTC in a GBM patient revealed by iFISH. CTC identified by SE-iFISH was FISH^+^ (polyploidy chromosome 8, orange, A) /GFAP^+ or –^ (green, B) /DAPI^+^ (blue, C) and CD45^−^ (red, D). **E.** Four image emerged. **F-K.** CTC detected in different pathologic types of brain gliomas. **(F):** anaplastic astrocytoma; **G.** Anaplastic oligodendroglioma; **H.** oligoastrocytoma; **I.** oligodendroglioma; **J.** anaplastic oligoastrocytoma; **K.** astrocytoma.

Figure 2A-E showed a circulating tumor cell enriched from peripheral blood of a GBM patient. As described previously, cancer cells were identified as non-hematopoietic (CD45-) and polyploidy of chromosome 8 by FISH.A large strong polyploidy (≥5 copies) chromosome 8^+^, CD45^−^ CTC was observed, with GFAP negative expressed. WBCs surrounding CTC were diploid of chromosome 8, and stained positively for CD45. Figure 2F-K showed CTC detected in different pathologic subtypes of gliomas. In our study, all the detected CTCs exhibited ≥ 5 copies of chromosome 8.

### Detection of CTC in different pathologic subtypes of gliomas

No cell containing polyploidy chromosome 8 in PB of the 10 healthy controls has been detected. The detailed clinical and tumor characteristics and CTCs count of all 31 patients were listed in Table 1.

**Table 1:** Detailed clinical and tumor information of 31 patients of primary gliomas

No.	Sex	Age	KPS	tumor region	Pathologic type	WHO grade	CTC counts	
							before operation	1week after operation
1	M	23	70	right frontoparietal	OA	2	0	not performed
2	M	37	80	left parietooccipital	AOA	3	1	not performed
3	M	25	90	right thalamic	AOA	3	0	not performed
4	F	48	90	right frontal	GBM	4	6	not performed
5	F	52	80	right frontal	AA	3	1	not performed
6	F	44	70	right parieto- temporooccipital	GBM	4	10	not performed
7	M	39	80	right fronto- temporoinsular	OA	2	1	not performed
8	F	66	80	left frontal	A	2	2	not performed
9	M	28	90	right frontoparietal	GBM	4	0	not performed
10	M	44	90	left fronto- temporoinsular	OA	2	2	not performed
11	F	44	80	left frontal	GBM	4	1	not performed
12	M	41	90	left temporo- hippocampal	AO	3	0	not performed
13	M	68	60	left cerebellar	GBM	4	0	not performed
14	F	58	90	left frontal	GBM	4	2	not performed
15	M	34	80	right frontal	O	2	6	not performed
16	F	45	90	right frontal	O	2	2	not performed
17	M	46	90	right fronto- temporoinsular	AO	3	6	not performed
18	F	16	90	left frontal	A	2	0	not performed
19	M	27	90	right frontal	AO	3	0	not performed
20	M	30	90	left frontoparietal	GBM	4	2	not performed
21	M	30	90	left frontoparietal	GBM	4	1	not performed
22	F	46	90	left occipital	AOA	3	3	1
23	F	35	90	right fronto- parietotemporal	astrocytoma	3	4	3
24	M	28	90	right temporal	OA	2	1	2
25	F	52	90	left frontal	GBM	4	2	11
26	M	53	80	right temporal	GBM	4	2	2
27	F	45	90	left fronto- parietotemporal	A	2	6	8
28	F	50	90	right temporal	GBM	4	5	4
29	F	44	90	left frontal	AA	3	1	0
30	M	41	90	right frontal	OA	2	7	6
31	F	30	90	left frontal	O	2	3	10

Abbreviations: A, astrocytoma; O, oligodendroglioma; OA, oligoastrocytoma; AA, anaplastic astrocytoma; AO, anaplastic oligodendroglioma; AOA, anaplastic oligoastrocytoma; GBM, glioblastoma multiforme; KPS, Karnofsky performance scale.

**Figure 3:**
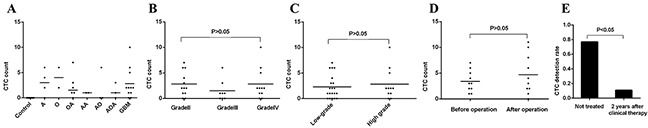
Comparison of CTC incidence and count **A.** CTC count in 7 pathologic types of gliomas. **B.** Comparison of CTC count in gliomas of grade II (n=12), grade III (n=8) and grade IV (n=11). **C.** Comparison of CTC count between low (n=20) and high grade (n=11). **D.** Comparison of CTC count in patients (n=10) before and after operation. **E.** Comparison of CTC incidence between patients not treated (n=31) and patients 2 years after standard clinical intervention (n=9). Note: “Not treated” = 31 primary glioma patients who did not receive any clinical intervention, including surgical intervention, chemotherapy and radiotherapy.

We found CTCs could be detected in all the 7 pathologic subtypes of gliomas. The CTC detection results of different pathologic subtypes of gliomas were shown in Figure 3A. Of the total 31 patients, the incidence of CTC was 77% (24/31), with CTC count varying from 1 to 10 per 7.5 ml PB. The CTC incidence in PB of patients with grade II, III and IV were 83%, 63% and 82%, respectively, and no statistically significant difference was observed (*P*=0.525). The incidence of CTC in low-grade glioma was 83%, with no statistically significant difference in high-grade glioma (74%, *P*=0.538). It was also noticed that no statistically significant difference of CTC count in patients with grade II (median 2, range 0-7), III (median 1, range 0-6) and IV (median 2, range 0-10) (*P*=0.303) (Figure 3B) or with low (median 1.5, range 0-7) and high grade (median 2, range 0-10) (*P*=0.690) (Figure 3C).

### Effects of clinical intervention on CTC incidence and count in glioma

To investigate whether hematogenous tumor cell dissemination was associated with tissue manipulation during surgical intervention, we selected 10 sequential patients with positive CTC in PB (patients 22-31) to detect the CTC count at 1 week post operation and for comparison with the pre-operation levels. The results revealed that in general, there was no significant difference (*P*=0.301) (Figure 3D) of CTC count in patients before (average 3.4±2.1) and after operation (average 4.7±3.9). But interestingly, we also noticed that 2 cases had dramatic increase of CTC count following surgical resection (Patient#25, 2 vs 11 and Patient#31, 3 vs 10, before and post operation respectively).

To further investigate whether these spreading CTCs could survive and proliferate for a long time, we selected 9 sequential patients to detect the CTC count who survived over 2 years after the standard clinical therapy. The detailed clinical characteristics of the 9 patients were listed in Table 2. The result showed there were significant decreases of CTC incidence (11% vs 77%, *P*<0.01) (Figure 3E) in patients 2 year after receiving clinical therapy. Of the 9 patients, only one patients was observed have one CTC (exhibited ≥ 5 copies of chromosome 8) in PB, and tumor recurrence in this patient was confirmed by operation 2 week later (data not shown).

**Table 2:** Clinical characteristics and CTC detection of patients 2 years after clinical therapy

NO	Age	Sex	Diagnosis	Surgery	Radiotherapy	Chemotherapy	KPS	Time after surgery(month)	CTC count
1	58	M	AOA	GTR	IMRT 5000cGy/25F	AVM	90	24	0
2	59	F	AOA	GTR	IMRT 6000cGy/30F	TMZ+AVM	70	24	0
3	32	M	A	GTR	IMRT 50.4Gy/28F	AVM	80	24	0
4	58	M	AA	GTR	IMRT 5000cGy/30F	AVM	50	26	0
5	55	F	OA	GTR	IMRT 5000cGy/28F	TMZ+AVM	90	26	0
6	41	M	A	GTR	-	TMZ+AVM	90	40	0
7	42	F	A	GTR	IMRT 6000cGy/30F	TMZ	90	45	1
8	21	M	A	STR	Gamma Knife 16Gy	-	90	90	0
9	53	M	O	GTR	IMRT 5000cGy/28F	TMZ	80	148	0

Abbreviations: A, astrocytoma; O, oligodendroglioma; OA, oligoastrocytoma; AA, anaplastic astrocytoma; AO, anaplastic oligodendroglioma; AOA, anaplastic oligoastrocytoma; GBM, glioblastoma multiforme; KPS, Karnofsky performance scale; IMRT, intensity modulated radiation therapy; TMZ, temozolomide; AVM, nimustine, vincristine and methotrexate.

### CTC could be used to distinguish tumor recurrence from radiation necrosis

**Figure 4:**
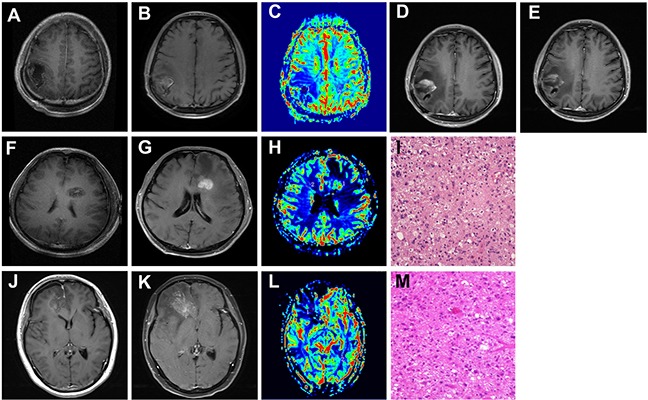
The clinical application of CTC in distinguishing tumor recurrence from radionecrosis **A, F, G.** Contrast axial T1-weighted image. After gross total resection, there is a surgical cavity without enhancement. **B, G, K.** Contrast axial T1-weighted image. After completion of RT, there is a new enhancing mass lesion on the initial post-RT MRI. **C, H, L.** rCBV map showed hypoperfusion (C and L, Patient 1 and Patient3) or hyperperfusion (H in patient 2) of the enhancing lesion (ROI 1) when compared to the contralateral normal white matter (ROI 2). **D.** and **E.** Follow-up of MRI performed 3(D) and 4(E) months after the initial post-RT MRI (Patient 1). **I, M.** Pathological findings in the second operation: tumor recurrence of GBM (Patient 2 and 3, HE, 10 x10). Patient 1: image A-E. Patient 2: image F-I. Patient 3: image J-M.

Given the high incidence of CTCs detected in different subtypes of primary gliomas, but decreased significantly a long time after standard clinical intervention, we attempted to investigate its clinical application in distinguishing tumor recurrence from radiation necrosis. We enrolled 5 patients harboring new enhancing mass lesion(s) on the initial post-RT MRI after the first gross total surgical resection. The clinical characteristics and CTC detection results of the 5 patients were listed in Table 3. Among the 5 patients, 2 displayed negative CTC inclining to radionecrosis (Patient 1 and patient 4), which were further confirmed in the reduced rCBV image of enhancement region, (Figure 4, patient 1, A-C). These two patients did not receive surgical operation but were followed up for the subsequent 3-4 months. In patient 4, no significant increase of enhancing lesion on MRI image was observed 3 months later, while the situation of patient 1 was more interesting. In patient 1, MRI scan was first performed 3 months later, but with an increased enhancing lesion that appeared like tumor recurrence. (Figure 4, patient 1, D). Since no clinical symptoms were observed in this patient, he did not receive any anti-tumor treatment but was asked to visit our clinic 1 month later for review. Very surprisingly, the MRI performed this time showed a significant decrease of enhancing lesion compared to 1 month ago (Figure 4, patient 1, E), confirming the initial diagnosis of radionecrosis based on the consistent results of CTC detection and MRI perfusion.

**Table 3:** Clinical characteristics and CTC detection of patients with tumor recurrence or radionecrosis

No.	Age	Sex	Diagnosis	Region	Surgery	CT	RT (IMRT)	month after RT	KPS	rCBV	CTC count
1	44	M	GBM	right parietal	STR	TMZ	60Gy/30Fr	5	90	0.56	0
2	45	M	GBM	left frontal	GTR	TMZ	60Gy/30Fr	13	90	2.38	2
3	46	F	AA	right frontal	GTR	TMZ	60Gy/30Fr	29	80	0.67	8
4	31	M	AOA	right frontal	GTR	TMZ	60Gy/30Fr	8	90	0.65	0
5	45	F	GBM	left parieto- occipital	GTR	TMZ	60Gy/28Fr	6	70	2.27	3

Abbreviations: CT, chemotherapy; RT, radiotherapy; AA, anaplastic astrocytoma; AOA, anaplastic oligoastrocytoma; GBM, glioblastoma multiforme; KPS, Karnofsky performance scale; IMRT, intensity modulated radiation therapy; TMZ, temozolomide.

Another 2 patients (patient 2 and patient 5) showed positive CTC concurrent with increased rCBV, which predicted tumor recurrence. The next reoperation pathological diagnosis had also proved it (Figure 4, patient 2, F-I). Noteworthy, the last patient exhibited positive CTC but reduced rCBV in MRI enhancement region, giving rise to diagnostic uncertainty of tumor recurrence or radionecrosis (Figure 4, patient 3, J-L). As a result of MRI image presented local midline shift that indicated cerebral hernia, the patient was performed operation after an informed consent obtained from family members. Consequently, the postoperative pathology confirmed that the patient suffered from tumor recurrence (Figure 4, patient 3, M).

## DISCUSSION

Considering the distinct internal environment of brain, Cushing and Bailey first proposed that glioma would never lead to extracranial metastases. However, in 1928, Davis for the first time fully reported cases of glioma concurrent with extracranial metastasis, posing an alert towards the ingrained viewpoint [19]. In the following years, experts have tried to find the clues of glioma migration, but no substantial progress obtained, because the detection of tumor cells from primary brain tumors presented a unique challenge. The first real breakthrough were the findings of CTCs in the PB of GBM patients [10, 20, 21]. It subverted the hypothesis “soil and seed” founded by Stephen Paget and provided a completely original research direction of glioma migration.

Since the detection of CTCs in the PB of glioma patients was at the initial stage, the technology of each institution adopted was significantly differed. The earliest method used to find CTCs was based on the detection of tumor cell surface specific markers such as EpCAM, cytokeratins (CKs) and glial fibrillary acidic protein (GFAP) etc. [10, 22–24]. Muller et al. identified CTCs in PB from 29 of 141 (20.6%) GBM patients by immunostaining of enriched mononuclear cells with antibodies directed against GFAP and stated that CTCs are “intrinsic property” of GBM biology [10]. However, GFAP expression is not totally restricted to glial cells [25, 26], and it was therefore important to exclude interference impact of a minor subpopulation of GFAP-positive cells derived from non-glial cells. In 2014, Macarthur et al. described a strategy to detect CTCs in patients with brain tumors based on telomerase activity, which is elevated in nearly all tumor cells except normal cells. They maintained that the telomerase-based system could offer high sensitivity (as greater than 90% of solid tumors, including glioma) and high specificity (as telomerase was epithelial cell independent) [20]. However, this telomerase-based strategy was relatively time-consuming and procedure-complicated. At approximately the same time, Sullivan et al. applied the CTC-iChip to test for the presence of GBM CTCs. Their results showed that CTCs were identified in at least one blood specimen from 13 of 33 patients (39%) [21]. Compared with the high expense of this approach, the CTCs detection rate seemed to be unsatisfied.

In this study, the methodology we applied for isolation, detection and identification of CTCs in the PB was reported by Ge et al. in 2015, which has successfully detected glioma tumor cells in CSF, based on polyploidy of chromosome 8 examination by CEP8-FISH [6]. We demonstrated that chromosome 8 polyploidy cells generally existed in 10 brain glioma specimens, which is consistent with the results in many other tissues, including lung [13], esophageal [14], pancreatic [15], gastric [16], colon [17], and bladder [18], etc. Until now, there is litter information about chromosome 8 polyploidy in gliomas. We noticed in a study that the frequency of polysomy chromosome 8 was higher in long-term survivors of GBM, with the highest frequency of 30-40% in a specific group having the longest survival times [27]. Since the significance of polysomy 8 have not been well studied in brain gliomas, especially in low-grade gliomas before, we suppose that it should be a quite new but very meaningful field to further explore its biological and clinical value in future.

In the present study, the overall incidence of CTCs in the PB of patients with glioma was 77%, which was consistent with MacArthur et al.'s result [20]. CTCs has been detected in all seven pathological subtypes of glioma, irrespective of different malignant degree, which suggested CTCs should be a common property of glioma rather than a property exclusive to GBM. To our knowledge, it was currently the report disclosing the highest CTCs detection rate in gliomas.

It provided an ultimate paradox in the hypothesis “soil and seed” [28]. As for the cause of CTCs entering into the circulation system, we initially hold that surgical intervention could promote cancer cell dissemination. Our results revealed that the CTCs counts of patients tested before or after operation were almost the same, which was in accordance with Muller's result [10]. However, we also noticed that 2 of the 10 cases, had significant increase of CTC count 1 week after the operation. Due to the small sample size, the clinical significance of these 2 cases could not be determined, and a larger sample study (both patients sample and more points of time after operation for CTC detection) should be included in future.

Until now, the mechanism of CTC escape from the blood–brain barrier has not been fully understood. Recently, it was reported that some specific characteristics of CTCs could be the reason for their more invasive property [21]. By using CTC-iChip for CTC detection in GBM, Sullivan et al reported that all of the GBM CTCs detected in patient samples shared a mesenchymal expression profile. Subsequent RNA in situ hybridisation (RNA-ISH) results demonstrated that sub-populations of primary GBM cells that express the high mesenchymal expression profile were more invasive to get into the bloodstream, which could help explain this phenomenon. However, it is noteworthy that a comprehensive and in-depth understanding of the mechanisms is still lacking, especially in gliomas of low WHO grades. Our results implicated that the ‘seed’ might disseminate via some other insidious approaches that are still unknown.

Another mysterious field of CTC in brain glioma is that although the glioma tumor cells could invade into bloodstream, they rarely turned into metastatic lesions in peripheral organs. Two points of view could be used to explain this phenomenon. First, the “soil” in the peripheral organs, was unsuitable for the development of glioma. Glioma cells usually require some critical neural-specific growth factors that are absent outside the brain. According to the findings of Dihua Yu et al. [29], tumor cell could normally express PTEN (an important tumor suppressor) in peripheral organs, but once transplanted into brain, the PETN expression reduced. Meanwhile, its expression would be restored to the previous level when the cell was separated from brain. Their study demonstrated that, in the brain, the exosomes secreted by neurogliocytes could create a unique “fertile soil” environment that was suitable for the growth of cancer “seed”. Second, the “seed”, namely CTCs, harbors some intrinsic changes that can restrict the extracranial metastasis of glioma. For instance, immune-mediated suppression of glioma cells carrying epitopes that are usually masked by the blood brain barrier, may underlie the general failure of glioma proliferation in peripheral organs [21]. Combined with our presented results, which confirmed that glioma cells of various pathological types could enter the circulation but rarely metastasis, it is suggested that in addition to the “seed”, the “common soil in brain” may be another field worthy of further research. However, all these hypotheses need further investigation in the future.

It is indisputable that the tradition monitoring for treatment response and progression in patients with glioma currently presents challenges [3, 30]. “Pseudoprogression” or radionecrosis may mimic progressive disease, which commonly results in the events of overtreatment and under treatment [4, 31]. As CTCs widely existing in the PB of patients with glioma, it serves as direct evidence of glioma extracranial migration. Our results showed the CTCs count of patients after chemotherapy and/or radiotherapy was significantly lower than those before adjuvant treatment, and the detection of CTCs could contribute to differentiating radionecrosis from true tumor progression. In this study, we discovered the CTCs detection results were always consistent with the MRI findings in both radionecrosis and progression. Most noteworthy, we encountered a patient with decreased rCBV region in the peritumor zone, but subsequent FISH detection showed 8 CTCs in the PB supporting the early finding of progression. The patient was later confirmed to have tumor recurrence by histopathology. Collectively, CTCs can be regarded as a complement of MRI and it, to some extent, is superior to MRI in monitoring the treatment response and local microenvironment of glioma.

There are, inevitably, some shortcomings in the present study, including our limited sample size and the absence of molecular biomarkers such as IDH1/2, TERT, MGMT which are of great clinical significance [32–34]. Since the short follow-up, the prognostic potential of CTCs detected in the primary brain gliomas cannot be disclosed at present. We believe that a larger sample size with more detailed data can provide more information about the application value CTCs detection. These efforts are currently being pursued in our ongoing studies.

In conclusion, the identification of CTCs serves as a common property of gliomas of various pathological subtypes, which has provided an ultimate paradox for the hypothesis “soil and seed”. CTCs can be used to monitor the microenvironment of gliomas dynamically, which will be a meaningful complement to radiographic imaging.

## MATERIALS AND METHODS

### Patients, pathological evaluations and ethics

A series of 10 healthy volunteers and 31 patients with newly diagnosed primary gliomas (16 males and 15 female), belonging to 7 different pathological subtypes, in Beijing Tiantan Hospital from December 2015 to February 2016 were enrolled in the present study. The patients were numbered 1 to 31 in the temporary order of testing.

To explore the short-term significance of surgical intervention on CTC count, we selected 10 sequential patients (patient 22-31) to detect the CTC count 1 week post-operation and for comparison with the pre-operation levels. To explore the long-term significance of clinical therapy (including surgery, chemotherapy and/or radiotherapy) on CTC count, we selected 9 sequential patients, who visited our outpatient clinic in Dec 2015 over 2 years after the standard clinical therapy, to detect the CTC count and for comparison with patients who did not accept clinical treatment.

To explore the clinical value of CTC for differentiating between radiation necrosis and tumor relapse, we selected 5 patients, who received radiotherapy after gross total surgical resection, as having new enhancing mass lesion(s) on the initial post-RT MRI. CTC detection and MRI perfusion was performed for differentiation of radionecrosis or tumor recurrence, and the diagnosis of tumor recurrence was at last determined based on pathologic analysis after the second resection. Patients diagnosed with radionecrosis were followed up, and the diagnosis of radionecrosis was made if no significant increase of enhancing mass lesion(s) was observed in 6 months, and during the follow-up period patients did not receive any anti-tumor treatment, except mannitol injection.

All patients were underwent chest X-ray examination and blood tests routinely before operation, patients with the abnormal results or patients with other diseases were excluded in our study. The study was approved by the Medical Ethics Committee of Beijing Tiantan hospital and written informed consent was obtained from all participants. To avoid bias, blood sample collection, enrichment, SE-iFISH and result reading were blindly performed by different personnel. The pathological result of all specimens was independently reevaluated by three experienced neuro-pathologists. In case of discrepancy, the three observers simultaneously reviewed the slides to achieve a consensus.

### Assessment of polyploid chromosome 8 statue in glioma specimen by FISH

Polyploid chromosome 8 was detected in 10 patients' tumor specimens with FISH, and tumor cell-free surrounding tissues evidenced under pathologic microscopy were selected from 10 patients as negative controls. The interpretation threshold was defined as mean+3x SD. Vysis CEP8 SpectrumOrange Direct Labeled Fluorescent DNA Probe kit was used for FISH test. 4 um thick paraffin slides were deparaffinized, dehydrated, and incubated in 1 mol/L NaSCN for 35 min at 80°C. Slides were then immersed in pepsin solution (0.65 % in protease buffer with 0.01 mol/L HCl) for 10 min at 37°C, and tissues were fixed by 10 % neutral buffered formalin. Then the specimens were dehydrated in ethanol (70, 85, and 100 %, 2 min in each bath) and air-dried. 20 ul of each probe was then added separately and slides were sealed with rubber cement. After co-denaturation for 10 min at 75°C, the slides were then put in a humidified atmosphere with Hybrite (ThermoBriteTM vysis) 16 h at 37°C. Slides were then immersed in 2x SSC/0.3 %NP-40 for 2 min at RT, and then in 2xSSC/0.3 % NP-40 for 2 min at 73°C. After drying, nuclei were counterstained with 4,6-diamidino-2-phenylindole (DAPI) and antifade compound (p-Phenylenediamine). FISH signals for each locus-specific FISH probe were assessed under an Olympus BX51TRF microscope (Olympus, Ina-shi, Nagano, Japan) equipped with a triple-pass filter (DAPI/Green/Orange; Vysis). The entire areas of the tissue microarray cores were evaluated in each case and as many non-overlapping nuclei as possible (≥100 per hybridization) were assessed for red (target) signals.

### CTC subtraction enrichment (SE)

CTC subtraction enrichment was performed as previously described [6] and experiment was performed according to the product manufacture's instruction (Cytelligen, San Diego, CA, USA). Briefly, 7.5 ml peripheral blood were collected and centrifuged at 800 × g for 7 min. All sedimented cells were loaded on the top of 3 ml of non-hematopoietic cell separation matrix, followed by centrifugation at 450 × g for 7min. Solutions above RBC were collected and incubated with 150 μl of anti-WBC and endothelial cell immunomagnetic beads for 15 min, followed by transferring to the top of the separation matrix. Samples were centrifuged at 450 × g for 7 min. Supernatants were collected and subjected to magnetic separation of beads. Bead-free solution was spun at 650 × g for 3 min. The resulting pellet containing rare cells was thoroughly mixed with 100 μl cell fixative, followed by application to the formatted and coated CTC slide (Cytelligen). Air dried samples are suitable for subsequent analyses, including immunofluorescent staining and iFISH described below.

### Immunostaining-FISH

The experimental procedure and CTC identification were conducted as previous described [6]. Experiment was performed according to the product manufacture's instruction (Cytelligen). Briefly, samples on the coated CTC slides were subjected to Vysis Centromere Probe (CEP8) SpectrumOrange (Abbott Laboratories, Abbott Park, IL, USA) hybridization for 90 min using a S500 StatSpin ThermoBrite Slide Hybridization/Denaturation System (Abbott Molecular, Des Plaines, IL, USA), followed by incubation with Alexa Fluor 594 conjugated monoclonal anti-CD45 and Alexa Fluor 488 conjugated with monoclonal anti-GFAP (BD) as described above. Images of the identified tumor cells were collected using a fluorescence microscope (OLYMPUS, BX-53) equipped with a filter set (Omega Optical, Brattleboro, VT, USA) for DAPI (Cytelligen), Alexa Flour 488 (BD), Alexa Flour 594 (Cytelligen), and Spectrum Orange, TRITC (Vysis). CTC is defined as DAPI+, CD45-, and polyploidy CEP8 signal with or without visible GFAP signal.

### MRI perfusion

MRI scans were obtained using 3.0-T magnets (Trio, SIEMENS, Germany). Conventional sequences were acquired using 5-mm slice thickness and 6-mm gap. DSC sequences were acquired using 5-mm slice thickness and 1.5-mm gap. DSC MRI was obtained using gradient-echo echo-planar images (repetition time/echo time=1400/32 ms, matrix 320×320, flip angle 90°, number of slices 19). The contrast bolus was injected though a peripheral Angiocath (22 gauge) using an MRI-compatible power injector at 5 ml/s and immediately followed by a 20-ml saline flush at the same rate. Multisection image data were acquired every second for a total of 75s, with the bolus contrast injection occurring after 10s. A standard dose (0.2 ml/kg of body weight, maximum dose 20 ml) of gadopentetate dimeglumine (BEILU Pharmaceutical CO.LTD, Beijing, China) contrast was administered for 3.0-T scans. DSC perfusion analysis was performed in consensus by a neuroradiology fellow and a radiology resident (with 5 and 3 years of experience in MRI perfusion, respectively) Analysis was performed while blinded to the outcome diagnosis of radionecrosis vs. tumor recurrence. Small, fixed diameter (50–100 mm^2^) regions of interest (ROIs) were placed over the enhancing mass lesion and compared with control ROIs placed over the contralateral normal-appearing white matter to calculate tumorous relative cerebral blood volume (rCBV). Blood vessels, cystic/necrotic changes, and areas of susceptibility from hemorrhage, bone, or air were excluded from the ROIs. The rCBV measurements were recorded as CBVlesion/CBVnormal-appearing white matter.

### Statistical analysis

SPSS version 22.0 was used for all statistical analyses. The Chi-square test was used to determine CTC incidence differences in 2-group comparison and multi-group comparison. The paired t-test was used to determine CTC count differences of patients pre- and post-operation. The Wilcoxon-test was used to determine CTC count differences in other 2-group comparisons and multi-group comparison. CTC count of different groups were presented as the mean±standard deviation(data obeyed normal distribution) or median with range(data not obeyed normal distribution). *P*-values <0.05 were considered to be statistically significant.

## References

[1] Morgan LL (2015). The epidemiology of glioma in adults: a “state of the science” review. Neuro-oncology.

[2] Wen PY, Reardon DA (2016). Neuro-oncology in 2015: Progress in glioma diagnosis, classification and treatment. Nature Reviews Neurology.

[3] Wen PY, Macdonald DR, Reardon DA, Cloughesy TF, Sorensen AG, Galanis E, DeGroot J, Wick W, Gilbert MR, Lassman AB (2010). Updated response assessment criteria for high-grade gliomas: response assessment in neuro-oncology working group. Journal of Clinical Oncology.

[4] Brandsma D, Stalpers L, Taal W, Sminia P, van den Bent MJ (2008). Clinical features, mechanisms, and management of pseudoprogression in malignant gliomas. The lancet oncology.

[5] Best MG, Sol N, Zijl S, Reijneveld JC, Wesseling P, Wurdinger T (2015). Liquid biopsies in patients with diffuse glioma. Acta neuropathologica.

[6] Ge F, Zhang H, Wang DD, Li L, Lin PP (2015). Enhanced detection and comprehensive in situ phenotypic characterization of circulating and disseminated heteroploid epithelial and glioma tumor cells. Oncotarget.

[7] Fonkem E, Lun M, Wong ET (2011). Rare phenomenon of extracranial metastasis of glioblastoma. Journal of Clinical Oncology.

[8] Kalokhe G, Grimm SA, Chandler JP, Helenowski I, Rademaker A, Raizer JJ (2012). Metastatic glioblastoma: case presentations and a review of the literature. Journal of neuro-oncology.

[9] Piccirilli M, Brunetto GMF, Rocchi G, Giangaspero F, Salvati M (2008). Extra central nervous system metastases from cerebral glioblastoma multiforme in elderly patients. Clinico-pathological remarks on our series of seven cases and critical review of the literature. Tumori.

[10] Müller C, Holtschmidt J, Auer M, Heitzer E, Lamszus K, Schulte A, Matschke J, Langer-Freitag S, Gasch C, Stoupiec M (2014). Hematogenous dissemination of glioblastoma multiforme. Science Translational Medicine.

[11] Albertson DG, Collins C, McCormick F, Gray JW (2003). Chromosome aberrations in solid tumors. Nature genetics.

[12] Kops GJ, Weaver BA, Cleveland DW (2005). On the road to cancer: aneuploidy and the mitotic checkpoint. Nat Rev Cancer.

[13] Cappuzzo F, Varella-Garcia M, Rossi E, Gajapathy S, Valente M, Drabkin H, Gemmill R (2009). MYC and EIF3H Coamplification significantly improve response and survival of non-small cell lung cancer patients (NSCLC) treated with gefitinib. Journal of thoracic oncology.

[14] Doak S, Jenkins G, Parry E, D'souza F, Griffiths A, Toffazal N, Shah V, Baxter J, Parry J (2003). Chromosome 4 hyperploidy represents an early genetic aberration in premalignant Barrett's oesophagus. Gut.

[15] Griffin C, Morsberger L, Hawkins A, Haddadin M, Patel A, Ried T, Schrock E, Perlman E, Jaffee E (2007). Molecular cytogenetic characterization of pancreas cancer cell lines reveals high complexity chromosomal alterations. Cytogenetic and genome research.

[16] Sánchez-Pérez I, Alonso PG, Iniesta CB (2009). Clinical impact of aneuploidy on gastric cancer patients. Clinical and Translational Oncology.

[17] Steiner MG, Harlow SP, Colombo E, Bauer KD (1993). Chromosomes 8, 12, and 17 copy number in Astler-Coller stage C colon cancer in relation to proliferative activity and DNA ploidy. Cancer research.

[18] Acar H, Kılınç M, Yıldırım MS, Kaynak M, Cenker A (2003). Evaluation of chromosome 8 and 11 aneuploidies in washings and biopsy materials of bladder transitional cell carcinoma. Cancer genetics and cytogenetics.

[19] Pasquier B, Pasquier D, N'golet A, Panh MH, Couderc P (1980). Extraneural metastases of astrocytomas and glioblastomas clinicopathological study of two cases and review of literature. Cancer.

[20] Macarthur KM, Kao GD, Chandrasekaran S, Alonso-Basanta M, Chapman C, Lustig RA, Wileyto EP, Hahn SM, Dorsey JF (2014). Detection of brain tumor cells in the peripheral blood by a telomerase promoter-based assay. Cancer Res.

[21] Sullivan JP, Nahed BV, Madden MW, Oliveira SM, Springer S, Bhere D, Chi AS, Wakimoto H, Rothenberg SM, Sequist LV (2014). Brain tumor cells in circulation are enriched for mesenchymal gene expression. Cancer discovery.

[22] Gires O, Stoecklein NH (2014). Dynamic EpCAM expression on circulating and disseminating tumor cells: causes and consequences. Cellular and Molecular Life Sciences.

[23] Weng Y-R, Cui Y, Fang J-Y (2012). Biological functions of cytokeratin 18 in cancer. Molecular Cancer Research.

[24] Woelfle U, Sauter G, Santjer S, Brakenhoff R, Pantel K (2004). Down-regulated expression of cytokeratin 18 promotes progression of human breast cancer. Clinical cancer research.

[25] Danielyan L, Tolstonog G, Traub P, Salvetter J, Gleiter CH, Reisig D, Gebhardt R, Buniatian GH (2007). Colocalization of glial fibrillary acidic protein, metallothionein, and MHC II in human, rat, NOD/SCID, and nude mouse skin keratinocytes and fibroblasts. Journal of Investigative Dermatology.

[26] Lim MCC, Maubach G, Zhuo L (2008). Glial fibrillary acidic protein splice variants in hepatic stellate cells-expression and regulation. Molecules and cells.

[27] Kim Y-W, Koul D, Kim SH, Lucio-Eterovic AK, Freire PR, Yao J, Wang J, Almeida JS, Aldape K, Yung WA (2013). Identification of prognostic gene signatures of glioblastoma: a study based on TCGA data analysis. Neuro-oncology.

[28] Fidler IJ (2003). The pathogenesis of cancer metastasis: the'seed and soil'hypothesis revisited. Nature Reviews Cancer.

[29] Zhang L, Zhang S, Yao J, Lowery FJ, Zhang Q, Huang W-C, Li P, Li M, Wang X, Zhang C (2015). Microenvironment-induced PTEN loss by exosomal microRNA primes brain metastasis outgrowth. Nature.

[30] Melguizo-Gavilanes I, Bruner JM, Guha-Thakurta N, Hess KR, Puduvalli VK (2015). Characterization of pseudoprogression in patients with glioblastoma: is histology the gold standard?. Journal of neuro-oncology.

[31] Clarke JL, Chang S (2009). Pseudoprogression and pseudoresponse: challenges in brain tumor imaging. Current neurology and neuroscience reports.

[32] Jiang H, Ren X, Cui X, Wang J, Jia W, Zhou Z, Lin S (2013). 1p/19q codeletion and IDH1/2 mutation identified a subtype of anaplastic oligoastrocytomas with prognosis as favorable as anaplastic oligodendrogliomas. Neuro-oncology.

[33] Jiang H, Ren X, Zhang W, Ma J, Sui D, Jiang Z, Cui X, Lin S (2013). A new prognostic scoring scale for patients with primary WHO grade III gliomas based on molecular predictors. Journal of neuro-oncology.

[34] Heidenreich B, Rachakonda PS, Hosen I, Volz F, Hemminki K, Weyerbrock A, Kumar R (2015). TERT promoter mutations and telomere length in adult malignant gliomas and recurrences. Oncotarget.

